# Oxidative Stress in Preterm Newborns

**DOI:** 10.3390/antiox10111672

**Published:** 2021-10-23

**Authors:** Chiara Lembo, Giuseppe Buonocore, Serafina Perrone

**Affiliations:** 1 Department of Molecular and Developmental Medicine, University of Siena, 53100 Siena, Italy; lmb.chr@gmail.com (C.L.); giuseppe.buonocore@unisi.it (G.B.); 2 Department of Medicine and Surgery, Neonatology Unit, University of Parma, 43126 Parma, Italy

**Keywords:** oxidative stress, free radicals, newborn infants, prematurity, antioxidants, biomarkers

## Abstract

Preterm babies are highly susceptible to oxidative stress (OS) due to an imbalance between the oxidant and antioxidant systems. The generation of free radicals (FR) induces oxidative damage to multiple body organs and systems. OS is the main factor responsible for the development of typical premature infant diseases, such as bronchopulmonary dysplasia, retinopathy of prematurity, necrotizing enterocolitis, intraventricular hemorrhage, periventricular leukomalacia, kidney damage, eryptosis, and also respiratory distress syndrome and patent ductus arteriosus. Many biomarkers have been detected to early identify newborns at risk of developing a free radical-mediated disease and to investigate new antioxidant strategies. This review reports the current knowledge on OS in the preterm newborns and the newest findings concerning the use of OS biomarkers as diagnostic tools, as well as in implementing antioxidant therapeutic strategies for the prevention and treatment of these diseases and their sequelae.

## 1. Introduction

The global incidence of preterm birth is approximately 10% of newborns [1] and represents the leading cause of neonatal mortality and morbidity [2]. Premature birth is referred to a birth occurring before 37 weeks of gestation and it is classified in four degrees in relation to gestational age of birth: extremely preterm (<28 weeks), very preterm (28–31 weeks), mild preterm (32–33 weeks), and moderate preterm (34–36 weeks) [3]. Spontaneous premature delivery without induction represents the 80% of all the preterm deliveries and the etiology still remains not fully understood [4].

Organ maturation, especially nervous system development, appears to be particularly intense during last weeks of pregnancy [5,6]. When a preterm birth occurs, the fetal growth must progress outside the womb. However, the new external environment is challenging for the newborns, especially if preterms encounter conditions to which they struggle to adapt [5,6]. In particular, preterms are highly susceptible to oxidative stress (OS), generated by an imbalance between oxidant and antioxidant that leads to an increased level of free radicals (FR) with subsequent oxidative damage to organs [7]. Moreover, the transition from low oxygen intrauterine (PO_2_ 100 mmHg) to a richer oxygen extrauterine environment (PO_2_ 20–25 mmHg) predispose to FR generation, exacerbated by the especially immature antioxidant system of the preterm [8]. Furthermore, oxygen resuscitation [9] and intensive care maneuvers such as assisted ventilation, surfactant administration [10], total parenteral nutrition [11], and blood transfusions [12] enhance FR production, which further increments OS. This process compromises irreversibly the nervous system development and generates organ damage, especially to kidney, ocular system, lung, and bowel.

## 2. Oxidative Stress from Pregnancy to Birth

OS occurs as a consequence of the homeostatic imbalance between oxidant production and intracellular antioxidant systems [13]. FR are molecules with unpaired electrons that include reactive oxygen species (ROS), reactive nitrogen species (RNS), and sulfur-centered radicals, collectively named oxidants. FR are produced by metabolic redox reaction, mainly in the respiratory chain, microsomal cytochrome P450, and immune response system as a consequence of endogenous and exogenous processes such as hypoxia, asphyxia, ischemia, ischemia-reperfusion, inflammation, hyperoxia, neutrophil and macrophage activation, mitochondrial dysfunction, and Fenton chemistry [14,15].

FR are highly unstable molecules and are thus prone to react with cellular components causing lipid peroxidation, protein, nuclear acids, polysaccharides damage, and compromising of cellular ion channel membrane activity. These mechanisms interfere with cellular and tissues functions leading to organ damage in the neonatal period [16]. The most common ROS include superoxide ion (O_2_^•−^), hydrogen peroxide (H_2_O_2_), hydroxyl radical (^•^OH), hydroperoxide (ROOH), and peroxyradical (ROO^•^). Nitric oxide (NO^•^) usually reacts with other ROS to form peroxynitrite (ONOO^−^), which is part of the RNS [4]. RNS are able to produce the same tissue damage of that caused by oxidation through the nitration reaction, the introduction of a nitrogen group into an organic compound [4].

Antioxidants, produced endogenously or assumed exogenously, are able to counterbalance FR production by either directly neutralizing or removing ROS/RNS and repairing or protecting from ROS/RNS-induced cellular damage. The most common antioxidants include enzymes superoxide dismutase (SOD) and catalase (CAT), glutathione peroxidase (GPX), vitamins (vitamins C and E), minerals, and small-molecule thiols such as glutathione (GSH) [13]. When the FR level exceeds the antioxidant capacity either for an augmented FR production or low antioxidant concentration, as occurs in premature babies, the consequent OS causes cellular and tissue functioning alterations [17].

Early in utero life is vulnerable to perturbation, and compelling evidence indicates that the fetal period of development is extremely sensitive to environmental cues. Insufficient fetal substrates determine permanent structural and physiological changes, thus leading to long-lasting consequences in postnatal life [18]. Placentation and fetal organogenesis are the critical stages potentially affected by OS during pregnancy. The alteration in cell number, clonal selection of cells, and epigenetics modification in gene expressions could be the mechanisms implicated in permanent changes. OS and the consequent rapid cell aging is an important initiating mechanism underlying the programming process due to suboptimal pregnancy conditions such as undernutrition, pre-eclampsia, diabetes, and prolonged exposition to stress and cortisol [16].

The impairment of fetal growth is another important aspect. Small for gestational age (SGA) and intrauterine growth restriction (IUGR) are often used as synonyms, although they reflect two different concepts. SGA denotes a neonate whose anthropometric variables are lower than a given threshold value computed on a set of infants of the same gestational age. SGA includes infants who have not achieved appropriate birth weight because of maternal, uterine, placental, and fetal factors. IUGR refers to a clinical and functional condition and denotes fetuses unable to achieve their own potential growth: a fetus with IUGR would have been larger, with no adverse environmental or genetic factors affecting the growth [19]. Many factors are likely to underlie such abnormal development including genetic makeup and an adverse intrauterine environment due to prenatal hypoxic/ischemic injury or inflammatory/infective insults. Intrauterine infection, especially chorioamniositis, is one major cause of spontaneous preterm labor and delivery and often occurs in symptom-free mothers. The placenta contributes to the physiological adaptation of the mother and the fetus during human gestation, and inflammation of the fetal adnexae is frequently found when there is a premature rupture of the membranes and a preterm delivery. The relationship among placental histopathology, fetal growth, and perinatal mortality and morbidity has been studied but remains to be clarified [20,21,22].

Histological placental findings of inflammatory changes and underperfusion lesions have been demonstrated to be linked to high levels of OS biomarkers in the cord blood [23]. Evidence over the past 15 years has shown that IUGR and pre-eclampsia are associated with abnormal remodeling of the spiral arteries, reduced placental perfusion, and loss of placental exchange surface decreasing the transfer of oxygen and essential substrates from mother to fetus. Feto-placental hypoxemia is also a specific trigger for an acute increase in FR. The link between FR generation and OS- and FR-mediated diseases in the perinatal period is quite complex, depending on the severity, intensity, and timing of asphyxia; the stage at which the oxygen insult occurs; the degree of maturity of the organs; the characteristics of the ensuing reoxygenation/reperfusion phase; and the capacity to counteract the injury [24,25].

Preterm babies are particularly vulnerable to OS injuries due to the high energy demand for their growth, the high concentration of non-protein-bound iron (NPBI), and the immaturity of antioxidant systems [26]. Antioxidant enzymes are induced in the last weeks of pregnancy as defensive mechanisms that prepare the neonate to the increase of oxygen pressure [27]. Therefore, plasma of preterms shows a reduced antioxidant profile, characterized by low levels of GPX, SOD, CAT, carotene, riboflavin, proteinase, vitamin E, selenium, copper, zinc, ceruloplasmin, transferrin, and other plasma factors [28]. Premature babies are highly exposed to OS counterbalanced by weak antioxidant defenses, and thus preterms’ immature organs are more susceptible to OS damage, especially retinas, lungs, brain, and intestine. Therefore, OS appears to be the main responsible of several diseases occurring in preterm babies. Those pathologies includes bronchopulmonary dysplasia (BPD), retinopathy of prematurity (ROP), necrotizing enterocolitis (NEC), intraventricular hemorrhage (IVH), periventricular leukomalacia (PVL), kidney damage, eryptosis, and also respiratory distress syndrome (RDS) and patent ductus arteriosus (PDA) [29,30,31,32,33].

## 3. Oxidative Stress Biomarkers: Biochemistry

**Figure 1:**
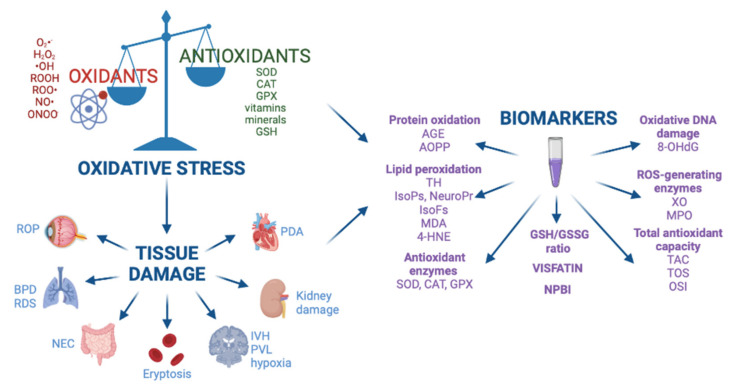
Oxidative stress, free radical-mediated diseases, and biomarkers. Abbreviations: BPD: bronchopulmonary dysplasia, ROP: retinopathy of prematurity, NEC: necrotizing enterocolitis, IVH: intraventricular hemorrhage, PVL: periventricular leukomalacia, RDS: respiratory distress syndrome, PDA: patent ductus arteriosus, AGE: advanced glycated end product, AOPP: advanced oxidation protein products, TH: total hydroperoxide, IsoPs: isoprostanes, IsoFs: isofurans, NeuroPr: neuroprostanes, MDA: malondialdehyde, 4-HNE: 4-hydroxy-2-nonenal, SOD: superoxide dismutase, CAT: catalase, GPX: glutathione peroxidase, GSH: glutathione, GSSG: glutathione disulfide, NPBI: non protein-bound iron, 8-OHdG: 8-hydroxy-2’-deoxyguanosine, XO: xanthine oxidase, MPO: myeloperoxidase, TAC: total antioxidant capacity, TOS: total antioxidant status, OSI: oxidative stress index. Image created in BioRender.com.

The role of OS in the pathogenesis of free radical-mediated diseases has been demonstrated by the dosage of biomarkers that evaluate and quantify the OS assessing the levels of by-products from proteins, lipids, and DNA damage (Figure 1). Biomarkers are divided in two categories, one reveals the potential risk of OS, such as NPBI, which generates ^•^OH through Fenton reaction; the other detects oxidation products of lipids, proteins, and DNA.

### 3.1. Lipid Peroxidation

Cellular membranes contain a high quantity of polyunsaturated lipids that are susceptible to FR-induced peroxidation, which leads to critical cell injury. This mechanism causes an irreversible cell injury that promotes the pathogenesis of free radical-mediated diseases. Total hydroperoxide (TH) measures the intermediate oxidative products of lipids and peptides, and thus it indicates the level of the overall OS. Isoprostanes (IsoPs) and neuroprostanes (NeuroPr) are produced from oxidation of arachidonic acid and docosahexaenoic acid, respectively. The IsoPs and NeuroPr are produced exclusively by free radical reactions and can be easily measured in plasma and urine [34]. Moreover, it has been demonstrated that there is an oxygen insertion step that diverts intermediates from the IsoPs pathway to instead form compounds, termed Isofurans, that contain a substituted tetrahydrofuran ring. This differential method of formation explains why oxygen tension can affect the lipid peroxidation profile. Like the IsoPs, the isofurans are chemically and metabolically stable and thus are well suited to act as in vivo biomarkers of OS. The ratio of isofurans to IsoPs also provides information about the relative oxygen tension in case of lipid peroxidation. Among IsoPs, the 8-iso-prostaglandin F2a (8-iso-PGF2a) has been associated with an increased risk of lung disease [35]. NeuroPr derive from FR peroxidation of docosahexanoic acid, a major component of neuronal membranes, and have been considered the best quantitative in vivo biomarker of oxidative damage in cerebral diseases. An alternative pathway of oxidation of docosahexanoic acid brings about the formation of isofuran-like compounds termed neurofurans, which are sensitive to changes in oxygen tension [26,36].

Malondialdehyde (MDA) is one of the most studied biomarkers of polyunsaturated fatty acid peroxidation, being highly cytotoxic because it is able to bind proteins or nucleic acids very quickly [26]. MDA is not an exclusive derivate of lipid peroxidation and furthermore is a minor secondary oxidation product; thus, the quantification of MDA should be interpreted with caution. The thiobarbituric acid reactive substances method of quantification has a poor specificity, but recently a new method of quantification at high resolution has been validated [37].

The reactive aldehyde 4-hydroxy-2-nonenal (4-HNE) is a reliable biomarker of lipid peroxidation that contributes as a nonclassical secondary messenger in several physiological processes at low concentrations, while it induces apoptosis and necrosis if it reaches supraphysiological values. In the condition of OS, it preferentially forms covalent bindings with cysteine residues of thiol-containing proteins involved in redox signaling, causing a disruption of the redox mechanism. It also affects glutathione metabolism and induces the production of H_2_O_2_ [38,39]. Furthermore, 4-HNE has been reported to interfere with the mitochondria activity by impairing ATPase function, altering oxygen consumption and promoting premature apoptosis [40].

### 3.2. Protein Oxidation

OS interferes with amino acid residues of proteins, leading to altered conformation, cross-linking, and loss of function. Oxidation of proteins implicates an introduction of carbonyl groups (-C=O) into the peptide sidechains forming carbonylated proteins. The “carbonyl stress” leads to the formation of AGE (advanced glycated end-product) molecules deriving from reduction reactions of sugars with amino groups of proteins, lipids, and DNA [41]. Thus, the measure of carbonyl levels reflects the OS-related protein damage. The advanced oxidation protein products (AOPP) in biologic fluids represent the result of plasma protein exposure to FR without oxidant properties; thus, the measure of AOPP estimates the OS-mediated protein damage [10].

### 3.3. Oxidative DNA Damage

8-Hydroxy-2’-deoxyguanosine (8-OHdG) is an oxidized nucleoside released upon repair of damaged DNA, used as a biomarker of OS-related DNA damage. 8-OHdG is excreted into the urine without being further metabolized; thus, urinary 8-OHdG is considered an important biomarker of cellular OS [42].

### 3.4. GSH/GSSG Ratio

The glutathione (GSH)/oxidized glutathione (GSSG) ratio regulates the cytoplasmic redox status balance, essential for the cellular metabolism. The GSH/GSSG ratio is considered one of the most valuable biomarkers to assess OS [43].

### 3.5. NPBI

Free iron catalyzes the Fenton reaction that produces the highly reactive radical ^•^OH from H_2_O_2_: Fe^2+^ + H_2_O_2_ => Fe^3+^ + OH + ^•^OH. Iron is stored in proteins such as hemosiderin and ferritin or bound to transport proteins. In physiological conditions, it takes part in aerobic metabolic processes, while it is toxic when unbound [44]. NPBI indicates a low molecular mass iron not bounded to plasma proteins, thus potentially available to react with H_2_O_2_ through the Fenton reaction to produce hydroxyl radicals [45]. In addition, it has been described that lipids exposed to high concentration of NPBI generate IsoPs [46].

### 3.6. ROS-Generating Enzymes: Xanthine Oxidase and Myeloperoxidase

ROS-generating enzymes such as xanthine oxidase (XO) and myeloperoxidase (MPO) are physiologically present in cells and take part to cellular metabolism but can also be found in the circulation. High circulating levels of XO and MPO may be involved in the OS burden in relation to substrates and the antioxidant conditions [47].

During hypoxia, the reduction of oxidative phosphorylation leads to rapid consumption of adenosine triphosphate (ATP), and adenosine byproducts are converted into hypoxanthine (Hx). XO catalyzes the oxidation of Hx to xanthine (Xa) and uric acid (UA), representing one of the major sources of FR in human physiology [48]. XO reaction leads to higher FR production in the reperfusion phase [10].

XO is present in two forms: the oxidase XO, which uses O_2_ to oxidize Xa to UA form H_2_O_2_, and the dehydrogenase XDH, which performs the same reaction with NAD+. NAD^+^ is an oxidizing agent that accepts electrons and becomes NADH, the reduced form of NAD^+^. During a hypoxic state, XDH is converted to XO, amplifying ROS generation [47,48].

MPO is a heme peroxidase that catalyzes the reaction between H_2_O_2_ and chloride ions with the production of HOCl as the primary oxidant. Some oxidation products are produced by MPO, such as 3-chlorotyrosine, an AOPP used as a biomarker [47,48].

### 3.7. Antioxidant Enzymes SOD, CAT, and GPX

SOD is one of the most important detoxification and antioxidant enzymes of the cellular system. It catalyzes the dismutation of two molecules of O_2_^•−^ into H_2_O_2_ and O_2_, neutralizing the superoxide anion harmful action [49]. Since SOD also produces the pro-oxidant product H_2_O_2_, other antioxidant enzymes such as CAT and GPX are needed [49].

CAT is an antioxidant enzyme that transforms H_2_O_2_ into H_2_O and O_2_ [50], completing the detoxification process initiated by SOD [49]. CAT is principally located in the peroxisomes but absent in the mitochondria, and therefore another enzyme known as GPX, mainly located in the mitochondria, is able to breakdown H_2_O_2_ to water and lipid peroxides to their corresponding alcohols through oxidation of GSH to GSSG [48,49].

### 3.8. Total Antioxidant Capacity

The total antioxidant capacity (TAC) or non-enzymatic antioxidant capacity indicates the moles of oxidants neutralized by one liter of body fluids. Non-enzymatic antioxidants in plasma are represented by endogenous compounds, such as uric acid, bilirubin, and thiols, and nutritional products, such as tocopherols, ascorbic acid, carotenoids, and phenolics [48]. The measure of TAC corresponds to the overall plasma antioxidant capacity that reflects the antioxidant status of the human body [51]. Similar to TAC, total antioxidant status (TOS) quantifies all oxidants in a specimen, and oxidative stress index (OSI) represents the ratio TOS/TAC [4].

### 3.9. Visfatin

Visfatin is a ubiquitous adipocytokine abundantly expressed in visceral fat, having been recently reported as a biomarker of inflammation and cellular dysfunction. Visfatin has also been described as a regulator of NAD^+^ metabolism. This multi-functional molecule has been related to OS, even if its pathophysiological role in humans still needs to be clarified [52]. A recent study by Marseglia et al. reported higher levels of visfatin in neonates more exposed to OS, possibly due to the high demand of NAD^+^. Therefore, visfatin has been proposed as a marker of OS for an early identification of neonates at high risk of OS-related damage. Consequently, visfatin may also be useful to identify subjects that could benefit from antioxidant treatments [53].

OS is defined as the homeostatic imbalance between oxidant production and antioxidant systems [13]. The most common ROS include O_2_^•−^, H_2_O_2_, ^•^OH, ROOH, ROO^•^, NO^•^, and ONOO^−^ [4]. Antioxidants such as enzymes SOD, CAT, GSH, GPX, vitamins, and minerals are able to counterbalance FR production [13]. When the FR levels exceeded the antioxidant capacity, the consequent OS causes organ disfunction, which determines several diseases including BPD, ROP, NEC, IVH, PVL, kidney damage, eryptosis, RDS, and PDA [17,29,30,31,32,33]. Biomarker dosage helps to evaluate and quantify the role of OS in the pathogenesis of free radical-induced pathologies. The main categories of OS biomarkers comprehend protein oxidation products, lipid peroxidation products, antioxidant enzymes, GSH/GSSG ratio, visfatin, NPBI, oxidative DNA damage derivates, ROS-generating enzymes, and total antioxidant capacity.

## 4. Oxidative Stress Biomarkers: Diagnostic Value

OS plays a fundamental role in the pathogenesis of the typical diseases of preterm infants, which can be demonstrated by the dosage of OS biomarkers. High levels of TH, AOPP, and NPBI detected in the cord blood have been related to an increased risk to develop a condition linked to potential free radical damage [44]. Thus, an early identification of OS-related biomarkers appears to be an important tool for the appropriate management of preterm newborns. In considerations of the multiple effects of OS, studies describe several biomarkers related to a single disease, grouped in a panel, which is useful in detecting the risk of developing a certain neonatal disease linked to OS [26] (Table 1).

The accumulation of oxidants in the early stage of prenatal life may represent a huge problem due to the potential effects of OS on proper fetal development and programming of adult diseases [16]. Fetal nutrient and oxygen availability depend on the rate of transfer across the “placental barrier”, which consists of two cell layers separating fetal and maternal circulations: the fetal capillary endothelium and the syncytiotrophoblast. The efficiency of transplacental exchange depends on a complex interplay that involves placental growth, transporter protein expression, rates of placental blood flow, transmembrane concentration gradients, and metabolic demands of the placental tissues. This interplay is orchestrated by the maternal, placental, and fetal hormones, and under favorable conditions ensures an adequate supply to the fetus [54]. OS has been presumed as one of the main risk factors of conditions associated with adverse pregnancy outcomes, such as pre-eclampsia, hypertension, diabetes, smoking, infection, or inflammation, as well as obesity and maternal malnutrition. Increased levels of IsoPs have been detected in the amniotic fluid of pregnant women who later experienced a IUGR and a preterm premature rupture of membranes (PROM). Hence, the assay of IsoPs in amniotic fluid can be considered a reliable assessment of fetal OS and a predictive index of risk of preterm PROM [16,21,22]. Furthermore, increased levels of OS biomarkers have been found in cord blood of neonates from obese mothers in comparison with lean controls [55]. Increased levels of MDA and lower TAC both in mothers and their SGA babies at birth compared to appropriate-for-gestational-age deliveries have been described [56]. At three days after labor, MDA and peroxides were found to be still higher with lower TAC in mothers who delivered SGA newborns compared to controls. High levels of 4-HNE-protein adducts were detected in the placenta stroma of SGA cases [56]. All these data suggest the relevant role of the placenta in the regulation of the oxidative status during labor and the potential relevance of OS in the pathophysiology of SGA.

Besides the direct action of FRs on cellular components, OS-related effects are mediated by perturbations on classical gene expression, on DNA repair regulation, and on epigenetic modification [57]. The mechanisms mediating epigenetic effects are DNA methylation and histone modification (acetylation and methylation) [58]. Epigenetic changes are directly linked to ROS, and the latter can be directly regulated by epigenetic mechanisms. In addition, direct chemical effects of ROS and reactive nitrogen species on nucleotides are known [59]. Moreover, epigenetics integrates microRNAs due to their capability to affect the methylation machinery and the expression of proteins involved in histone modifications. In turn, the expression of certain miRNAs is controlled by DNA methylation and chromatin modifications [60]. Prominent miRNAs are also regulated by OS, and they can act in a redox-sensitive manner, thus allowing adjustment of their action to the cellular redox state or disease-associated OS condition [61]. As a combinatorial approach, these mechanisms may then determine gene expression and the resultant phenotype, thus setting an “in utero programming” [18].

In preterm hypoxic newborns, higher Hx, Xa, UA, TH, and AOPP levels have been detected, and the increase of AOPP concentration has been reported to correlate with the degree of hypoxia. These findings strongly suggest that hypoxia positively correlates with the reactive oxygen metabolite production and OS damage [10,62].

Red blood cells in the perinatal period are a target of extracellular FR that increase hemolysis and, consequently, bilirubin production. The molecular mechanisms of the suicidal death of erythrocytes, named eryptosis, involve the susceptibility of neonatal erythrocytes to OS. Erythrocytes are also themselves generators of FR through the Fenton reaction, which maintains and amplifies the process [63].

The role of OS has been reported also in preterms affected by RDS: plasma levels of MDA, protein carbonyls, AOPPs, 8-OHdG, H_2_O_2_, and oxidant/antioxidant ratio, calculated as protein carbonyls/(superoxide dismutase + glutathione peroxidase), showed a significant increase in neonatal RDS compared to healthy preterm controls [64,65,66,67], furtherly augmented after three days of life [67]. In a recent study, it has been reported that OS biomarker levels correlated with the severity of RDS: AOPPs and 8-OHdG levels on day-0 and day-3 were significantly augmented in neonates with RDS grades III and IV compared with RDS grade II preterms [46].

OS is also involved in the development of BPD in the preterm newborn [68,69]. An increase of OS pulmonary biomarkers, such as protein carbonylation, and a decrement of antioxidant levels have been described in preterms who later developed BPD and who underwent mechanical ventilation with high oxygen requirement, compared to preterms who did not develop BPD and who required lower oxygen [70,71]. Higher concentration of MDA has been detected in the epithelial lining fluid of oxygen-dependent preterm infants who subsequently developed BPD [72]. Higher urinary levels of 8-OHdG were reported in preterm babies who later developed BPD compared to non-BPD infants [73]. Recently, a higher concentration of 8-OHdG in serum and tracheal aspiration samples was identified on the first day after birth and subsequently at day 28 in a cohort of BPD preterms compared to a non-BDP group. Thus, DNA damage in the respiratory tract caused by OS appears to be involved in BPD occurrence and high levels of 8-OHdG found in TA showed a good predictivity on BPD development [74]. Therefore, RDS and BPD preterm categories have both showed higher oxidation of lipids, proteins, and DNA compared to healthy neonates, suggesting OS cellular damage contributes to the impairment of lung growth and development [75].

OS has been found to be involved also in the pathogenesis of NEC. Higher TOS and OSI have been detected in plasma of preterm with NEC compared with controls. Furthermore, TOS and OSI levels seem to be correlated to NEC severity [76]. In addition, AOPP, TH, and NPBI levels in the cord blood were higher in babies who later developed NEC compared to controls [77]. Thus, OS biomarkers measure in cord blood might identify high risk for NEC, but still no unique biomarker has been identified as a diagnostic test useful for clinical practice [77,78].

In preterm babies, OS plays a relevant role in the post-ischemic kidney damage. The increase of alpha-1 microglobulin and *N*-acetyl-b-d-glucosaminidase, which are markers of tubular disfunction, has been related to high AOPP and TH levels in the first two weeks of life, suggesting an OS-induced kidney damage [79].

PDA represents a prematurity complication that can increase the risk of developing other conditions strictly related to OS such as BPD, kidney damage, NEC, and IVH [80]. It has been described that urinary IsoPs levels decreased 12–24 h after pharmacological PDA closure with ibuprofen and subsequently increased after seven days from treatment [81]. These findings suggest that ibuprofen has an antioxidant capacity to scavenge FR [82], which causes the initial decrease of OS biomarkers and a successive increase associated with the end of the drug effect [81]. A study by Demir et al. reported that a hemodynamically significant PDA increases oxygen requirement, thus inducing ROS synthesis. The mean pre-therapy TOS level and OSI value of the patients with hemodynamically significant PDA were significantly higher compared to controls [83]. After PDA treatment, no differences of mean values of TOS, TAC, and OSI were found compared to controls, and thus PDA closure might imply an antioxidant effect [83]. Inayat et al. described lower SOD activity, urinary catalase, plasma, and urinary 8-isoPGF2α in PDA preterms compared to healthy controls. While plasma 8-isoPGF2α levels rebounded after PDA closure, probably due to augmented oxygenation, plasma SOD has been found to be reduced after PDA treatment. In this context, SOD appears to regulate antioxidant functional ductus arteriosus closure. In fact, a low SOD capacity may lead to lower H_2_O_2_ synthesis, a critical component of ductus arteriosus closure, thus contributing to PDA persistence [84]. A recent study by Coviello et al. found that urinary IsoPs levels measured in the second day of life appear to be highly predictive of a hemodynamically significant PDA development, with a sensitivity of 82% and a specificity of 73%. Early detection of urinary IsoPs has been suggested to be implemented for clinical practice as a reliable biomarker of hemodynamically significant PDA, together with echocardiography findings [85].

Among the free radical-mediated diseases, the IVH together with diffuse white matter injury and punctate white matter lesions represent the most common causes of brain injury in preterm infants [86]. Different biomarkers have been detected to reveal the presence of the OS damage of the premature brain. High TH, AOPP, and NPBI cord blood levels in premature babies have been associated with the development of all grades of IVH. It has been suggested that these biomarkers are strictly related to FR production in the central nervous system as a consequence of OS damage [44]. High levels of NPBI have been detected in cerebrospinal fluid of premature babies who have experienced post-hemorrhagic ventricular dilatation [87]. Furthermore, NPBI has been described as the best early predictive biomarker of neurodevelopmental outcome [31]. IsoPs have also been reported as reliable biomarkers of brain OS damage, and increased levels of IsoPs in preterm infants have been associated with poorer neurodevelopmental outcomes at 12 months of corrected age [88]. OS has been described to be among the main responsible factors for the development of white matter injury in premature infants [89]. A recent study described an augmented adenosine (Ado) plasma level concentration at day 15 in premature babies, significantly associated with brain white matter lesions evidenced using MRI [90]. Ado represents a promotor of oligodendrocyte maturation [91], and it has been reported to be elevated in response of OS generation in preterms [92]. Therefore, Ado has been proposed as a biomarker to predict premature brain injury in preterm infants, but further studies are needed to evaluate its potential clinical usage [90]. Recently, a higher concentration of plasma IsoPs has been reported in infants who developed a white matter injury at term equivalent age. Hence, plasma IsoP measurement is proposed to be an early biomarker with a good sensitivity to identify newborns at risk for brain injury [93].

ROP is a disease that affects the retina bloodstream, occurring mainly in low birth weight preterm infants [94]. OS has been shown to be involved in the pathogenesis of ROP [10,95]. A series of biomarkers have been investigated for early detection and treatment monitoring of ROP, among which MDA [95], GSH/GSSG [96], 8-OHdG [97], and polyunsaturated fatty acid oxidative products seem to be the most valuable for clinical practice [98].

**Table 1:** Oxidative stress biomarkers in clinical studies on preterm newborns.

Diseases	Biomarkers	Population	Biological Specimen	References
Preterm PROM	IsoPs	16 pregnant women	Amniotic fluid	[21]
IUGR	IsoPs	37 pregnant women	Amniotic fluid	[22]
Hypoxia	Hx, TH, AOPP	34 hypoxic preterms	Cord blood, venous blood sample	[10]
	Hx, Xa, UA, TH, AOPP	39 hypoxic preterms	Cord blood, venous blood sample	[62]
RDS	MDA, protein carbonyl, 8-OHdG, low TAS	16 RDS preterms	Cord blood	[64]
	Protein carbonyl, ox/antiox ratio	37 RDS preterms	Venous blood sample	[65]
	MDA, H_2_O_2_, low CAT and SOD	31 RDS preterms	Venous blood sample	[66]
	AOPP, MDA, 8-OHdG, low TAC	40 RDS preterms	Venous blood sample	[67]
BPD	Protein carbonyl	61 BPD preterms	Tracheal aspiration	[70]
	MDA	10 BPD preterms	Epithelial lining fluid	[72]
	8-OHdG	60 BPD preterms	Urine	[73]
	8-OHdG	26 BPD preterms	Venous blood sample, tracheal aspiration	[74]
NEC	TOS, OSI	41 NEC preterms	Venous blood sample	[76]
	AOPP, TH, NPBI	29 NEC preterms	Cord blood	[77]
PDA pre-therapy	IsoPs	43 hsPDA preterms	Urine	[81]
	TOS, OSI	37 hsPDA preterms	Venous blood sample	[83]
	Low SOD, catalase, 8-isoPGF2α, TAS,	53 hsPDA preterms	Urine, venous blood sample	[84]
	IsoPs	60 hsPDA preterms	Urine	[85]
IVH	TH, AOPP, NPBI,	33 IVH preterms	Cord blood	[44]
	IsoPs	44 IVH preterms	Cord blood, venous blood sample	[93]
PHVD	NPBI	20 PHVD preterms	Cerebrospinal fluid	[87]
Poor neurodevelopmental outcomes	NPBI	384 preterms	Cord blood	[31]
	IsoPs	121 preterms	Venous blood sample	[88]
ROP	TOS, MDA	18 preterms	Venous blood sample	[95]
	GSH/GSSG	8 ROP preterms	Cord blood, venous blood sample	[96]
	8-OHdG, MDA	25 ROP preterms	Venous blood sample, urine	[97]

Abbreviations: 8-OHdG: 8-hydroxy-2’-deoxyguanosine, AOPP: advanced oxidation protein products, BPD: bronchopulmonary dysplasia, GSH/GSSG: glutathione/glutathione disulfide, hsPDA: hemodynamically significant PDA, Hx: hypoxanthine, 8-isoPGF2α: iso-prostaglandin F2, IsoPs: isoprostanes, IUGR: intrauterine growth restriction, IVH: intraventricular hemorrhage, MDA: malondialdehyde, NEC: necrotizing enterocolitis, NPBI: non protein-bound iron, OSI: oxidative stress index, PDA: patent ductus arteriosus, PHVD: post-hemorrhagic ventricular dilatation, PROM: premature rupture of membranes, RDS: respiratory distress syndrome, ROP: retinopathy of prematurity, SOD: superoxide dismutase, TAC: total antioxidant capacity, TAS: total antioxidant, TOS: total antioxidant status, TH: total hydroperoxide, UA: uric acid, Xa: xanthine.

## 5. Pain and Oxidative Stress in Preterm Neonates

Premature babies are able to perceive pain since nociceptive pathways develop by the 24th week of gestation. On the other hand, descending inhibitory circuits and dorsal horn synaptic connectivity are not functional until the 48th week of gestation. Moreover, neurotransmitters, which modulate pain, are not plenty available until the 40th week [99,100]. As a consequence, preterms experience more intense responses to pain stimuli due to the immature pain modulation and a lower threshold of pain compared to full-term babies [101]. In addition, preterm babies are exposed to more painful procedures related to the longer stay in neonatal intensive care unit. The repeated afferent nociceptive activity leads to an increase of FR production, which cannot be counterbalanced by the immature antioxidant system of preterm newborns [102].

Painful procedures induce autonomic responses such as tachycardia and decrease of oxygen saturation, resulting in a high energy requirement and oxygen consumption [103]. The increased utilization of ATP causes a higher breakdown into purine byproducts that contain adenosine converted into Hx, Xa, and UA, precursors of ROS [48]. ROS are able to induce central sensitization and activate cyclooxygenase enzymes with prostaglandin production, thus amplifying pain perception [104].

Increased OS biomarkers have been detected in preterm and full-term neonates undergoing painful procedures compared to those not exposed [102,105,106]. Bellieni et al. described a rapid increase of TH and AOPP in newborns after a painful procedure such as heel prick [105]. A study by Slater et al. observed an increase in MDA in preterm babies exposed to procedural pain, significantly correlated with heart rate elevation and reduced oxygen saturation [106]. A recent study by Perrone et al. confirmed a significant increase in AOPP blood concentration in response to painful stressors in the newborn [102].

Furthermore, OS has been related with higher behavior pain scores subsequent to a tissue-damaging procedure [107].

It has been demonstrated that early exposure of premature infants to painful stressors is related to brain dysmaturation, which is highly predictive of neurodevelopmental impairments [108]. In addition, the frequent experience of premature babies of procedural pain has been related to reduced brain volumes and functional connectivity, associated with poor functional and behavioral outcomes in childhood and early adolescence [109,110].

## 6. New Perspectives on Old Problems: Antioxidant Strategies in Clinical Trials

Many novel antioxidant strategies have been investigated for the treatment of OS damage in preterm newborns [111].

Melatonin is described as an effective antioxidant agent that is able to counteract FR-induced injury [112] due to the antioxidant and anti-inflammatory properties assessed in many clinical studies [113,114]. Melatonin has also been proposed as a potential tool to prevent BPD onset in mechanically ventilated newborns [115], as a neuroprotective agent for premature brain injury [116], and for the management of newborns with NEC [117].

Many antioxidant therapies have also been studied in order to manage respiratory complications of prematurity. In this context, supplementation or overexpression of antioxidant enzymes, such as endotracheal administration of recombinant human SOD, vitamins A and E, and surfactant replacement, have been described to exert a potential protective effect against FR-induced lung damage [111,118].

The erythropoiesis-stimulating agent administration in preterm babies, in particular erythropoietin, have been reported to have neuroprotective properties that prevent inflammation and promote neurogenesis and angiogenesis [119,120]. Erythropoietin administration was proven to be safe in preterm infants, not affecting mortality rate and major adverse effect occurrence in the short and long term [121,122,123]. Recently, Jakab et al. highlighted an improved white matter development and a weak but widespread effect in the overall structural connectivity network after erythropoietin treatment [124].

Resveratrol, epicatechin, and *N-*acetylcysteine were studied in the pathogenesis of retinal neovascularization, and their application in preterm infants has shown beneficial effects in the prevention of ROP [125,126]. Moreover, vitamins E and C, d-penicillamine, intratracheal recombinant human SOD, and allopurinol have been studied as therapeutic strategies and for the prevention of ROP [127,128,129]. Literature data have suggested that an optimal nutritional support, especially in regard to lipids and total calories, as well as an adequate weigh gain, contribute to reduce severe ROP incidence [130,131,132].

Lutein is a carotenoid with natural antioxidant properties, contained in human milk and present at high concentrations in the retina and macula. Recently, it has been suggested that lutein has a protective role against lipid peroxidation and photo-oxidation occurring in the retinal tissue [131]. Yet, there is still no strong evidence about the usage of lutein in ROP prevention, and further studies are needed [128,129].

Low levels of IGF-1 have been found in preterm babies with impaired brain growth, ROP, and other morbidities; thus, a supplementation with recombinant human IGF-1 and its binding protein IGFBP-3 has been proposed as a potential strategy to reduce ROP occurrence and other preterm birth morbidities [133].

Other antioxidant agents have been used for neuroprotection purposes such as vitamins C and E, inhibitors of nitric oxide synthase, allopurinol, albumin, docosahexaenoic acid, deferoxamine, prostaglandin inhibitors, magnesium sulfate, *N*-acetylcysteine, melatonin, lutein, and omega-3 polyunsaturated fatty acid [134].

A recent study described the role of caffeine as a therapeutic agent with antioxidant, anti-inflammatory, and anti-apoptotic properties that are able to perform neuroprotective functions on the developing brain of premature babies [135].

It has been reported that sensorial saturation, used as a form of analgesia during heel prick, significantly reduces pain and the consequent pain-related OS in newborn [102].

A study by Forde et al. provided preliminary evidence that preterm infants treated with kangaroo mother care in the neonatal intensive care unit setting exhibited reduced OS levels, measured through urinary allantoin, which has been proposed as a noninvasive biomarker of OS [136].

## 7. Conclusions

The existence of a redox homeostasis is essential for normal health and survival of the cell. OS occurs when there is an unbalance between pro-oxidant and antioxidant factors; this process leads to cellular and tissue damage. During the perinatal period, OS can be magnified by other predisposing conditions, such as hyperoxia, hypoxia, ischemia, hypoxia–reperfusion, and inflammation. Fetuses and newborns are particularly susceptible to OS and damage due to high oxygen consumption, weak antioxidant systems, and the inability to induce antioxidant defenses during the hyperoxic challenge at birth.

Although much progress has been made in the identification of the risk to develop free radical-mediated diseases, no specific antioxidant or anti-inflammatory therapeutic schemes are currently used in the neonatal setting.

The challenge for the future is to introduce a panel of OS biomarkers to identify newborns at high risk of oxidative damage early on; to improve the management of preterm babies; and to implement novel, tailored intervention programs.

Moreover, developing antioxidant strategies to effectively combat OS damage appears to be necessary. Finally, promoting an optimal environment to minimize stress will not only ensure optimal fetal development but will also reduce the risk of free radical-mediated damage to developing tissues and organs.

## Abbreviations

4-HNE	4-hydroxy-2-nonenal.
8-OHdG	8-hydroxy-2’-deoxyguanosine.
8-iso-PGF2a	8-iso-prostaglandin F2a.
Ado	adenosine
AGE	advanced glycated end product.
AOPP	advanced oxidation protein products.
ATP	adenosine triphosphate.
BPD	bronchopulmonary dysplasia.
CAT	catalase.
FR	free radicals.
GSH/GSSG	glutathione/glutathione disulfide.
GPX	glutathione peroxidase.
H_2_O_2_	hydrogen peroxide.
Hx	hypoxanthine.
IsoPs	isoprostanes.
IUGR	intrauterine growth restriction.
IVH	intraventricular hemorrhage.
MDA	malondialdehyde.
MPO	myeloperoxidase.
NEC	necrotizing enterocolitis.
NeuroPr	neuroprostanes.
NPBI	non-protein-bound iron.
OS	oxidative stress.
OSI	oxidative stress index.
PDA	patent ductus arteriosus.
PROM	premature rupture of membranes.
RDS	respiratory distress syndrome.
ROP	retinopathy of prematurity.
SGA	small for gestational age.
SOD	superoxide dismutase.
TAC	total antioxidant capacity.
TOS	total antioxidant status.
TH	total hydroperoxide.
UA	uric acid.
Xa	xanthine.
XO	xanthine oxidase.
